# Multipotent adult progenitor cells for hypoxic-ischemic injury in the preterm brain

**DOI:** 10.1186/s12974-015-0459-5

**Published:** 2015-12-23

**Authors:** Reint K. Jellema, Daan R. M. G Ophelders, Alex Zwanenburg, Maria Nikiforou, Tammo Delhaas, Peter Andriessen, Robert W. Mays, Robert Deans, Wilfred T. V. Germeraad, Tim G. A. M. Wolfs, Boris W. Kramer

**Affiliations:** School of Mental Health and Neuroscience (MHENS), Maastricht University, Universiteitssingel 40, Maastricht, 6229 ER The Netherlands; Department of Pediatrics, Maastricht University Medical Center, PO Box 5800, Maastricht, 6202 AZ The Netherlands; Department of Pediatrics, Máxima Medical Center, PO Box 90052, 5600 PD Veldhoven, The Netherlands; Department of Biomedical Engineering, Maastricht University, PO Box 616, Maastricht, 6200 MD The Netherlands; School for Cardiovascular Diseases (CARIM), Maastricht University, PO Box 616, Maastricht, 6200 MD The Netherlands; Regenerative Medicine, Athersys, Inc., 3201 Carnegie Avenue, Cleveland, OH 44115-2634 USA; School of Oncology and Developmental Biology (GROW), Maastricht University, Universiteitssingel 50, Maastricht, 6229 ER The Netherlands; Department of Internal Medicine, Division of Hematology, Maastricht University Medical Center, PO Box 5800, Maastricht, 6202 AZ The Netherlands

**Keywords:** Preterm, Hypoxic-ischemic encephalopathy, Multipotent adult progenitor cells, Neuroprotection

## Abstract

**Background:**

Preterm infants are at risk for hypoxic-ischemic encephalopathy. No therapy exists to treat this brain injury and subsequent long-term sequelae. We have previously shown in a well-established pre-clinical model of global hypoxia-ischemia (HI) that mesenchymal stem cells are a promising candidate for the treatment of hypoxic-ischemic brain injury. In the current study, we investigated the neuroprotective capacity of multipotent adult progenitor cells (MAPC^®^), which are adherent bone marrow-derived cells of an earlier developmental stage than mesenchymal stem cells and exhibiting more potent anti-inflammatory and regenerative properties.

**Methods:**

Instrumented preterm sheep fetuses were subjected to global hypoxia-ischemia by 25 min of umbilical cord occlusion at a gestational age of 106 (term ~147) days. During a 7-day reperfusion period, vital parameters (e.g., blood pressure and heart rate; baroreceptor reflex) and (amplitude-integrated) electroencephalogram were recorded. At the end of the experiment, the preterm brain was studied by histology.

**Results:**

Systemic administration of MAPC therapy reduced the number and duration of seizures and prevented decrease in baroreflex sensitivity after global HI. In addition, MAPC cells prevented HI-induced microglial proliferation in the preterm brain. These anti-inflammatory effects were associated with MAPC-induced prevention of hypomyelination after global HI. Besides attenuation of the cerebral inflammatory response, our findings showed that MAPC cells modulated the peripheral splenic inflammatory response, which has been implicated in the etiology of hypoxic-ischemic injury in the preterm brain.

**Conclusions:**

In a pre-clinical animal model MAPC cell therapy improved the functional and structural outcome of the preterm brain after global HI. Future studies should establish the mechanism and long-term therapeutic effects of neuroprotection established by MAPC cells in the developing preterm brain exposed to HI. Our study may form the basis for future clinical trials, which will evaluate whether MAPC therapy is capable of reducing neurological sequelae in preterm infants with hypoxic-ischemic encephalopathy.

## Background

Hypoxia-ischemia (HI) in the developing brain is strongly correlated with mortality and neurological morbidity in preterm and full-term infants, resulting in an enormous physical, psychological, and economic burden [1]. Unfortunately, no therapeutic cure is available for hypoxic-ischemic brain injury in preterm infants.

In a translational animal model of global HI in the preterm ovine fetus, our group has demonstrated that cell-based therapy may be a promising neuroprotective strategy for preterm neonates suffering from HI-induced brain injury [2]. We showed that intravenous administration of exogenous mesenchymal stem cells (MSC) protected against functional loss and structural injury of the preterm brain [2]. These neuroprotective effects were largely attributable to attenuation of (neuro) inflammatory processes, as indicated by decreased microglial activation and proliferation in the preterm brain and induction of peripheral T cell tolerance, which was associated with reduced cerebral infiltration of these immune effector cells [2].

Multipotent adult progenitor cells (MAPC), which are adherent bone marrow-derived cells of an earlier developmental stage than MSC have a high expansion potential, and their immunological properties make it possible to use them as a universal allogeneic donor [3–5]. Pre-clinical animal studies have demonstrated that in comparison to MSC, MAPC cells have stronger anti-inflammatory effects and are more potent in promoting endogenous tissue regeneration after ischemic and traumatic injury to the CNS [6–11]. In addition, the smaller size of MAPC cells compared to MSC facilitates passage through the pulmonary capillary bed, which may increase availability of MAPC cells in the systemic and cerebral vasculature and thus enhancing their neuroprotective effect [12].

Based on these superior qualities of MAPC, we aimed to assess the anti-inflammatory and neuroprotective potential of MAPC cells in the preterm brain exposed to global HI. We hypothesized that systemic administration of MAPC cells would attenuate cerebral and peripheral inflammation and prevent structural and functional brain injury after global HI in the preterm ovine fetus. We tested our hypothesis in a well-established pre-clinical animal model of preterm global HI. In this model, preterm ovine fetuses were exposed to global HI by transient umbilical cord occlusion (UCO) at 0.7 gestation which is equivalent to 30–32 weeks human gestation [13] followed by systemic administration of MAPC therapy during a 7-day reperfusion period.

The anti-inflammatory effects of MAPC cells were assessed in the preterm brain by analysis of microglial responses in the hippocampus and subcortical white matter. The spleen was assessed, since previous reports suggest that MAPC-induced alterations in the splenic inflammatory response may be responsible for their neuroprotective effect after brain injury [9]. Structural effects of systemic MAPC administration were assessed by histological white matter injury examination. Brain function was studied by analysis of cortical function by means of electrographic seizure activity and central (brain stem) function by means of baroreflex sensitivity.

## Methods

### Study approval

The experimental protocol and study design were in line with the institutional guides for animal experiments and were approved by the institutional Animal Ethics Research committee of Maastricht University, The Netherlands.

### Randomization and blinding

**Fig. 1:**
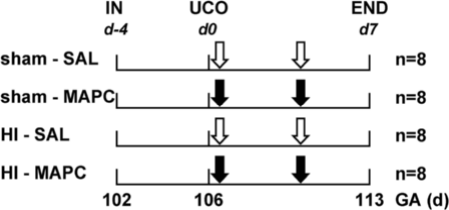
Study design. Fetuses were instrumented at a gestational age (GA) of 102 days. After a recovery period of 4 days, fetuses were subjected to 25 min of umbilical cord occlusion (UCO) or sham. One hour and 4 days after UCO or sham, fetuses received either intravenous MAPC (10 million cells, *closed arrow*) or saline 0.9 % (*open arrow*). After a 7-day reperfusion period, brain tissue was collected. Abbreviations: *in* instrumentation, *HI* hypoxia-ischemia, *SAL* saline, *MAPC* multipotent adult progenitor cells

Thirty-two singleton fetuses of time-mated Texel ewes were randomized by an independent researcher who was not involved in the animal experiments. Randomization resulted in four experimental groups: (1) sham umbilical cord occlusion, saline treatment (sham-SAL *n* = 8), (2) sham umbilical cord occlusion, MAPC treatment (sham-MAPC; *n* = 8), (3) umbilical cord occlusion, saline treatment (HI-SAL; *n* = 8), and (4) umbilical cord occlusion, MAPC treatment (HI-MAPC; *n* = 8) (Fig. 1). The investigator performing the (sham) umbilical cord occlusions was blinded to treatment allocation. Tissue sampling and the analyses of brain tissue and electrophysiological data were conducted in a blinded fashion.

### Animals and surgery

Singleton fetuses were surgically instrumented at 102 days gestational age (term ~147 days) as described previously [14]. Briefly, fetuses were partially exposed through a midline incision. A polyurethane umbilical vessel catheter (1.2 mm, Covidien, Mansfield, MA, USA) was placed in the left femoral artery and brachial vein for blood pressure recordings, blood sampling, and administration of MAPCs. An additional catheter was placed into the amniotic sac for measurement of amniotic fluid pressure. Three electrocardiogram (ECG) electrodes were placed on the fetal thorax for cardiac monitoring. Two pairs of custom-made shielded silver-tipped electroencephalogram (EEG) electrodes (Cooner wire Co., Chatsworth, CA, USA) were placed bilaterally on the dura over the parasagittal parietal cortex (with a subcutaneous silver reference electrode placed in the neck). An inflatable vascular occluder (OC16HD, 16 mm, In Vivo Metric, Healdsburg, CA, USA) was placed around the umbilical cord for induction of transient global HI. All fetal leads were exteriorized through the maternal flank. Post-operatively, sheep were housed individually with access to water and food ad libitum. A period of 4-day post-operative recovery was incorporated before onset of the experiments.

### Experimental design

**Fig. 2:**
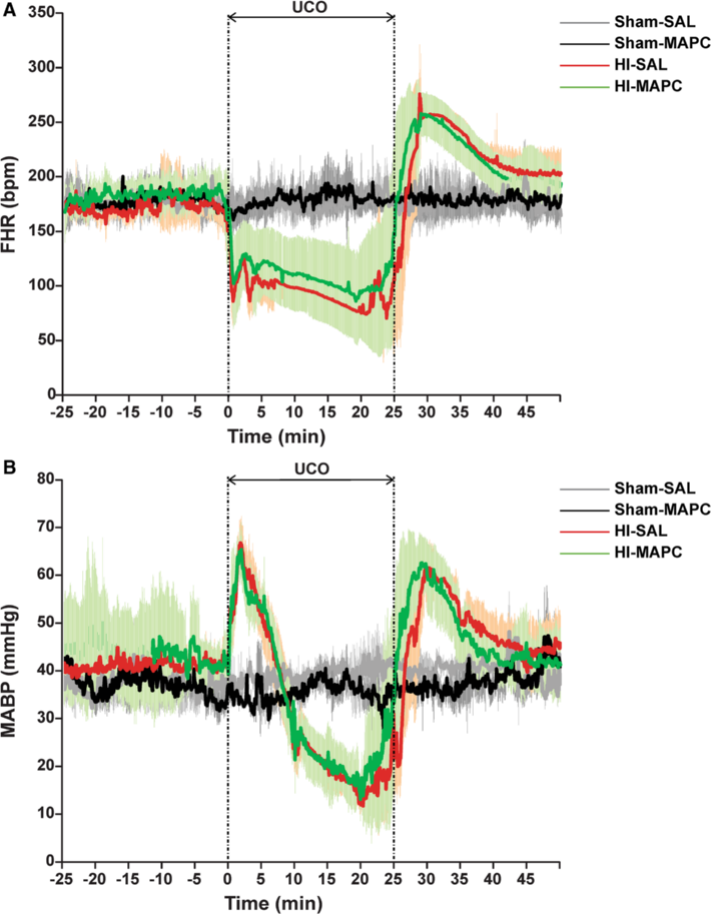
Reproducibility of 25 min umbilical cord occlusion (UCO) as evidenced by comparable vital parameters and blood gasses in animals exposed to global HI. Fetal heart rate (FHR) (**a**) and fetal mean arterial blood pressure (MABP) (**b**) measurements indicated that all animals exposed to global HI experienced the same degree of bradycardia and hypotension, respectively; means (*thick line*) ± SD (*shaded areas*) of *n* = 8 animals per experimental group are shown. *HI* hypoxia-ischemia, *SAL* saline, *MAPC* multipotent adult progenitor cells, *min* minutes

At 106 days gestational age (experimental day 0) fetuses were subjected to 25 min of (sham) umbilical cord occlusion by rapidly inflating the occluder with sterile saline of a defined volume known to completely inflate the occluder. An acute drop in heart rate and a gradual decline in blood pressure confirmed complete occlusion of the umbilical cord (Fig. 2). Global hypoxia-ischemia was further monitored with subsequent arterial blood gas analysis indicating acidemia, hypoxemia, and hypercapnia (data not shown). One hour after umbilical cord occlusion and on experimental day 4, fetuses received either an intravenous bolus of MAPC cells or an equal volume of vehicle. At the end of the experiment (experimental day 7), both ewe and fetus were euthanized by administration of pentobarbital (200 mg/kg).

### Data acquisition and analysis

Physiological data was sampled as described previously on a custom-made MPAQ unit (Maastricht-Programmable AcQuisition system, Maastricht Instruments BV, Maastricht, The Netherlands) with IDEEQ software (Maastricht Instruments BV) [14]. In short, EEG data were sampled at 1000 Hz and stored on a hard disk for off-line analysis. EEG data were filtered using a 0.5–30 Hz fourth order Butterworth band-pass filter. EEG signal with an amplitude >1000 μV was considered an artifact and removed from analysis (<1 % of data). After filtering, the raw EEG signals of the central and posterior channels were converted into amplitude-integrated EEG (aEEG) traces, using an (Matlab® based) algorithm similar to the clinical EEG NicoletOne™ device (Viasys Healthcare, Conshohocken, PA, USA), as previously described [2, 15–18]. EEG seizure activity was annotated manually using aEEG/EEG traces, and the total number and length of seizures was subsequently calculated. A neonatologist experienced in aEEG interpretation, who was blinded to treatment allocation, performed annotation.

ECG and blood pressure data were processed off-line using MATLAB R2013a (The MathWorks, Inc., Natick, MA). The ECG recordings were used to determine R-R intervals. Blood pressure measurements were corrected for amniotic pressure and used to determine the mean arterial blood pressure in each pressure wave. As previously reported, heart rate and heart rate variability were assessed in 192 s segment by calculating the mean R-R interval and standard deviation (SD-RR), respectively [19, 20]. Likewise, mean systolic blood pressure and blood pressure variability was assessed by calculating the mean SBP and standard deviation (SD-SBP) [19]. As a time series estimate of baroreflex sensitivity, we calculated the ratio between SD-RR and SD-SBP [19, 21, 22].

### MAPC

MAPC cells were provided by Athersys, Inc. (Cleveland OH, USA) and stored in liquid nitrogen. Cells were prepared and genomic and epigenetic stability of the cells were confirmed as previously described [5, 23]. Flow cytometric analysis of cell-surface proteins demonstrated that the cells were CD29- and CD90-positive, and MHC class I low, and were negative for MHC class II, CD45, CD106, and the costimulatory molecules CD80 and CD86. Cells were able to differentiate into mesenchymal cell types (adipocytes, osteoblasts, chondrocytes, and smooth muscle cells), but also toward endothelium [4]. Prior to injection, MAPC cells were thawed and washed twice and suspended in phosphate buffered saline (PBS) at a concentration of 10 × 10^6^ cells/mL. MAPC cells were administered intravenously 1 h and 4 days after (sham) UCO. Fetuses received two doses of 10 × 10^6^ cells in 1 mL PBS.

### Immunohistochemistry brain

After 3 months of submersion fixation in 4 % paraformaldehyde, the right cerebral hemisphere was embedded in gelatin, and serial coronal sections (50 μm) were cut on a Leica VT 1200S vibrating microtome (Leica Biosystems, Nussloch, Germany). Free-floating sections at the level of mid-thalamus and posterior hippocampus were stained with a rabbit anti-ionized calcium-binding adaptor molecule 1 (IBA-1) antibody (Wako Pure Chemical Industries, Osaka, Japan) for microglia or a rat anti-myelin basic protein (MBP) antibody (Merck Millipore, Billerica, MA, USA) for myelin sheaths and myelin-producing (mature) oligodendrocytes, and cleaved Caspase-3 for apoptosis (Cell Signaling Technology, Boston, USA) as previously described [14, 24].

Briefly, endogenous peroxidase-activity was blocked by incubation with 0.3 % H_2_O_2_ in tris-buffered saline (TBS, pH 7.6). Free-floating sections were incubated overnight at 4 °C with the diluted primary antibody (1:1000 anti-IBA-1, 1:2000 MBP, 1:800 Caspase-3) followed by incubation with the appropriate secondary antibodies. The immunostaining was enhanced with Vectastain ABC peroxidase Elite kit (PK-6200, Vector Laboratories, Burlingame, CA, USA) followed by a nickel sulfate-diaminobenzidine (IBA-1) or 3,3′-diamniobenzidine staining (MBP, Caspase-3). Sections were mounted on gelatin-coated glass slides, air-dried, dehydrated in ascending ethanol concentrations, and cover-slipped with PerTex.

### Analysis of immunohistochemistry

Immunohistochemical stainings were analyzed as previously described [14, 24]. For the analysis of IBA-1 and MBP immunoreactivity, digital images of the subcortical white matter (SCWM) (×100 magnification) and hippocampus (×20 magnification; only IBA-1) were acquired using an Olympus AX-70 microscope (Olympus, Tokyo, Japan) equipped with a black and white digital camera. In the hippocampus, area fraction of IBA-1 immunoreactivity was assessed in one digital image per section by delineating the hippocampus and determining the areal fraction of IBA-1 immunoreactivity expressed as a percentage of total hippocampal area with a standard threshold using Leica Qwin Pro V 3.5.1 software (Leica, Rijswijk, The Netherlands). In the SCWM, the area fractions of IBA-1 and MBP immunoreactivity were determined in five adjacent ×100 digital images obtained in standardized locations within the SCWM of each section. The results of five images per section were averaged to obtain the areal fractions of IBA-1 and MBP immunoreactivity within the SCWM for each section.

For analysis of cleaved Caspase-3, five adjacent digital images (×100 magnification) of the SCWM were obtained in standardized locations for each section. Numbers of Caspase-3-positive cells were determined with Leica Qwin Pro V 3.5.1 software and averaged to obtain cell numbers for Caspase-3 per section.

### RNA extraction and quantitative real-time PCR

For real-time (RT) quantitative polymerase chain reactions (qPCR), total RNA was extracted from spleen using the SV Total RNA isolation system (Z3100; Promega, Madison, WI, USA) according to the manufacturer’s recommendations. RT-qPCR reactions were performed in duplicate with the SensiMix SYBR No-ROX kit (QT650-02; Bioline Reagents Ltd) in a LightCycler 480 Instrument with ovine-specific primers for interleukin IL-10 (5′-CATGGGCCTGACATCAAGGA-3′ (sense), 5′-CGGAGGGTCTTCAGCTTCTC-3′ (antisense), TNFα (5′-GCCGGAATACCTGGACTATGC-3′ (sense), 5′-CAGGGCGATGATCCCAAAGTAG-3′ (antisense), and IFNγ (5′- TCAAGCAAGACATGTTTCAGAAGTTCT-3′ (sense), 5′-CCGGAATTTGAATCAGCCTTTTGAA-3′ (antisense). RT-qPCR results were normalized to the housekeeping gene ovine 40S ribosomal protein S15 (ovRPS15) (5′-CGAGATGGTGGGCAGCAT-3′ (sense), 5′-GCTTGATTTCCACCTGGTTGA-3′ (antisense)).

### MAPC detection

Detection of MAPC was performed with a PCR for human β-2 microglobulin [2]. Genomic DNA was isolated from snap frozen brain, spleen, and lung tissues using the Wizard Genomic Purification Kit (Promega, Leiden, The Netherlands) according to the manufacturer’s recommendations. PCR reactions of genomic DNA were performed for β-2 microglobulin followed by a nested PCR for β-2 microglobulin on the amplification product of the first PCR.

The amplified products were separated on ethidium bromide-stained 2 % agarose gels and captured using the Imagemaster® VDS equipped with a CCD camera (GE Healthcare Life Sciences (Pharmacia Biotech), Uppsala, Sweden). Serial dilutions of MAPC cells were used as references.

Primers used for the amplification of β-2 microglobulin were 5′- GTGTCTGGGTTTCATCAATC-3′ (sense), 5′- GGCAGGCATACTCATCTTTT-3′ (antisense), 5′- TGGGTTTCATCAATCCGACAT-3′ (nested sense) and 5′- CTCATCTTTTTCAGTGGGGGT-3′ (nested antisense).

### Statistics

Summary statistics of animal characteristics and all outcome variables are shown as means with 95 % confidence interval (CI). Groups’ comparisons of all outcome parameters were drawn with analysis of variance (ANOVA) or with random intercept models in case of repeated measurements per animal (e.g., different sections per brain). HI (sham vs. HI) and treatment (saline vs. MAPC) were the fixed effects. For random intercept models, animals constituted additionally the random effect. Variables, whose distributions were positively skewed, were log-transformed previous to statistical testing. To facilitate interpretation, averages on the log scale were back transformed to the original scale (antilog) and were presented as geometric means and corresponding 95 % CIs.

Seizure data, measured over time, showed pronounced right-skewness (caused by the absence of seizures in non-hypoxic conditions) that could not be remedied by log transformation. Hence, for these variables, pair-wise groups’ comparisons were performed with nonparametric Mann-Whitney tests, per individual time-point. They are presented as medians and corresponding interquartile range (IQR). A false discovery rate (FDR) of 5 % was used for multiple testing corrections. Groups’ differences with FDR corrected *P* < 0.05 were considered statistically significant. Statistical analysis was performed with IBM SPSS Statistics Version 20.0 (IBM Corp., Armonk, NY, USA).

Statistical analysis of baroreflex data was performed using a Bayesian multi-level model [25] to estimate the daily effects of global HI and MAPC treatment under hypoxic and sham conditions on the baroreflex sensitivity. This model corrects for repeated measurements and takes into account variance between and within subjects [25]. Statistical time series analysis was conducted using the Stan for R package (version 2.6.0) in R (version 3.1.1).

## Results

### Animal characteristics

To test the neuroprotective potential of MAPC therapy, we randomized 32 preterm sheep fetuses in four different experimental groups (Fig. 1). After instrumentation and a recovery period of 4 days, animals were subjected to 25 min of (sham) umbilical cord occlusion (UCO) to induce global hypoxia-ischemia (HI). MAPC cells were administered 1 h and 4 days after UCO (Fig. 1). Fetal body weight and gestational age did not differ significantly between the four experimental groups (Table 1). In line with previous reports [2, 14, 15], the fetal response to hypoxia-ischemia was comparable in all animals exposed to UCO (Fig. 2), indicating that the injury group (HI-SAL) and intervention group (HI-MAPC) were exposed to a similar degree of global hypoxia-ischemia.

**Table 1:** Animal characteristics

	sham-SAL	sham-MAPC	HI-SAL	HI-MAPC
GA at UCO (days)	105.2 (105.0;105.5)	106.0 (105.3;106.7)	105.5 (105.1;106.0)	105.2 (104.9;105.8)
BW (g)	1690 (1516;1865)	1532 (1245;1820)	1542 (1362;1721)	1559 (1340;1779)
Brain weight (g/kg BW)	17.58 (16.04;19.12)	17.39 (14.72;20.05)	15.97 (14.43;17.51)*	16.08 (14.37;17.80)

Fetuses were subjected to umbilical cord occlusion (UCO) at a comparable gestational age (GA). Fetal body weight (BW) did not differ between experimental groups. Brain weight corrected for body weight was significantly reduced following global HI. MAPC treatment did not prevent loss of cerebral weight. Means ± 95 % CI are shown. Groups’ comparisons were drawn with ANOVA (sham-SAL *n* = 8, sham-MAPC *n* = 8, HI-SAL *n* = 8, HI-MAPC *n* = 8)

*P ≤ 0.05

*HI* hypoxia-ischemia, *SAL* saline, *MAPC* multipotent adult progenitor cells

### MAPC reduced the cerebral inflammatory response after global HI

**Fig. 3:**
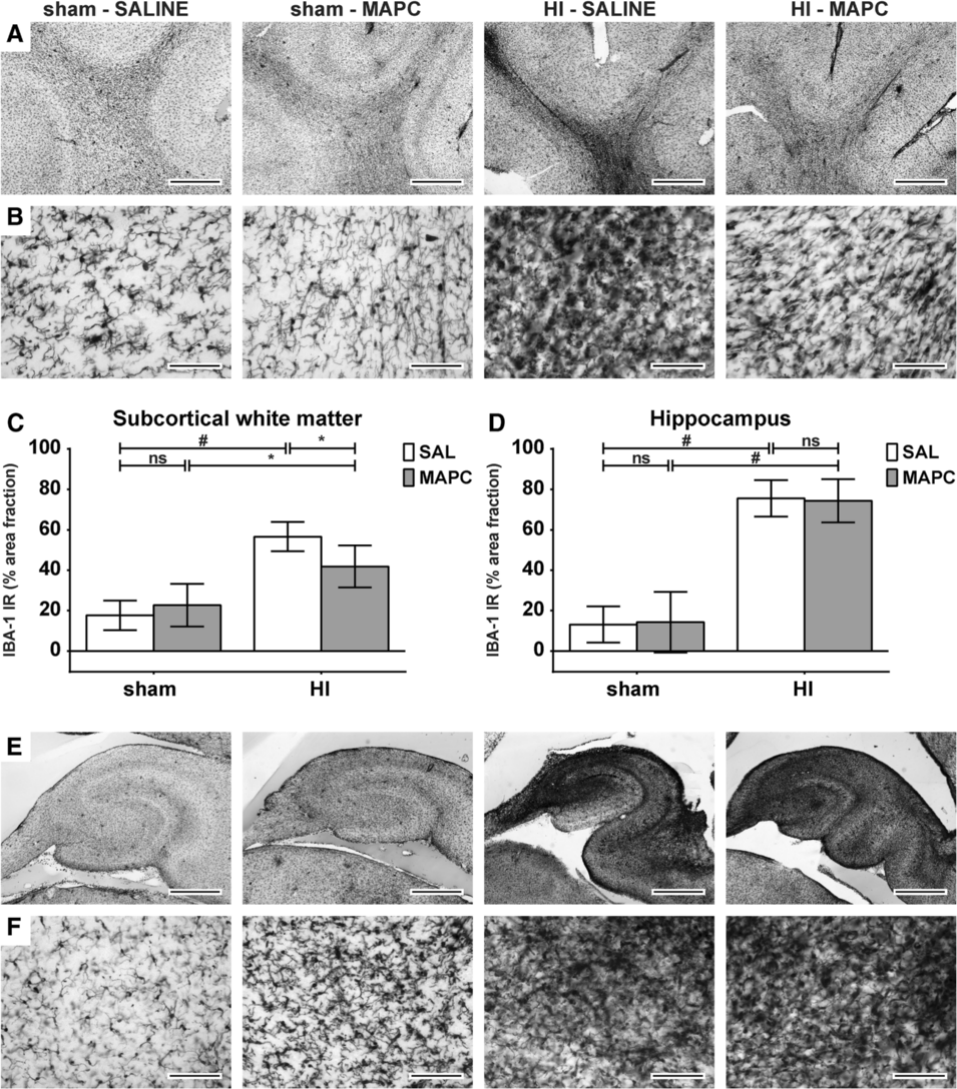
MAPC reduced cerebral inflammation in the subcortical white matter (SCWM), but not in the hippocampus. **a**–**b** Immunohistochemical IBA-1 staining in the SCWM at ×20 (**a**) and ×200 (**b**) magnification and hippocampus of the four experimental groups (sham-SAL *n* = 8, sham-MAPC *n* = 5, HI-SAL *n* = 8, HI-MAPC *n* = 5). Global HI induced a profound increase of IBA-1 immunoreactivity and amoeboid morphology in both regions, which was significantly reduced by MAPC in the SCWM, but not in the hippocampus. **c**–**d** Graphical presentation of area fraction of IBA-1 immunoreactivity in SCWM and hippocampus. Geometric means ± 95 % CI and levels of significance are depicted, which were calculated by the random intercept model with all repeated measures (i.e., brain sections) per animal. **e**–**f** Immunohistochemical IBA-1 staining in the hippocampus at ×20 (**e**) and ×200 (**f**) magnification of the four experimental groups. IBA-1 IR was markedly increased and accompanied by amoeboid morphology following global HI, which remained unaffected after MAPC treatment (sham-SAL *n* = 8, sham-MAPC *n* = 5, HI-SAL *n* = 8, HI-MAPC *n* = 5). *P ≤ 0.05, §*P* ≤ 0.01, #*P* ≤ 0.001. *IBA-1* ionized calcium-binding adaptor molecule 1, *HI* hypoxia-ischemia, *SAL* saline, *MAPC* multipotent adult progenitor cells, *IR* immunoreactivity. **a**–**b** Scale bars: **a**–**e** represent 1 mm, **b**–**f** 100 μm

The cerebral inflammatory response was studied by assessing microglial proliferation in the subcortical white matter and hippocampus using ionized calcium-binding adaptor molecule 1 (IBA-1), which is a highly-specific marker for resting and activated microglia in sheep [14]. Global HI resulted in a significant (sham-SAL vs. HI-SAL; *P* < 0.001) increase of IBA-1 immunoreactivity in the SCWM and hippocampus, indicating profound microglial proliferation in these regions (Fig. 3a–f). MAPC significantly (HI-SAL vs. HI-MAPC; *P* = 0.043) reduced IBA-1 immunoreactivity in the SCWM (Fig. 3a–c). MAPC did not reduce IBA-1 immunoreactivity in the hippocampus (HI-SAL vs. HI-MAPC; *P* = 0.881) (Fig. 3d–f). No differences in IBA-1 immunoreactivity were observed between saline or MAPC-treated sham-operated animals in the SCWM and hippocampus.

### MAPC prevented HI-induced white matter injury

**Fig. 4:**
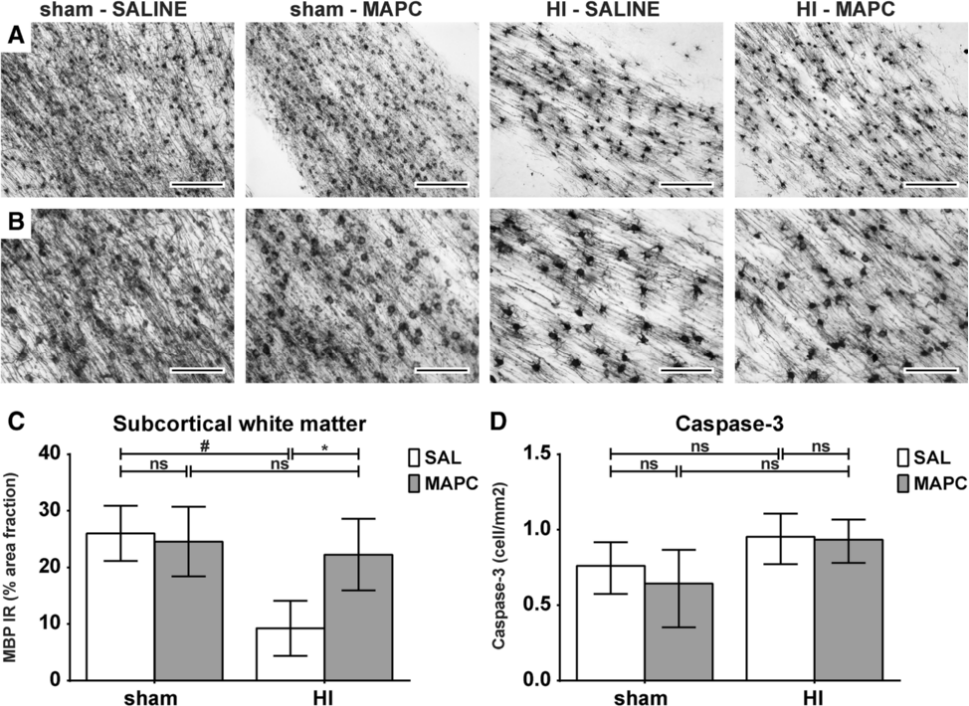
MAPC reduced white matter injury after global HI. **a**–**b** Immunohistochemical MBP staining in the SCWM of the four experimental groups at (**a**) ×100 and (**b**) ×200 magnification. **c** MBP immunoreactivity in SCWM. Global HI induced marked hypomyelination in the SCWM. MAPC significantly prevented the decrease in MBP reactivity after global HI. The area fraction of MBP was similar in sham conditions. Geometric means ± 95 % CI and levels of significance are depicted, which were calculated by the random intercept model with all repeated measures (i.e., brain sections) per animal (sham-SAL *n* = 8, sham-MAPC *n* = 5, HI-SAL *n* = 8, HI-MAPC *n* = 5). **d** Numbers of Caspase-3-positive cells in the four experimental groups; geometric means ± 95 % CI and levels of significance are depicted, which were calculated with ANOVA (sham-SAL *n* = 8, sham-MAPC *n* = 8, HI-SAL *n* = 8, HI-MAPC *n* = 8). *P ≤ 0.05, §*P* ≤ 0.01, #*P* ≤ 0.001. *MBP* myelin basic protein 1, *HI* hypoxia-ischemia, *SAL* saline, *MAPC* multipotent adult progenitor cells, *IR* immunoreactivity. Scale bars: (**a**) 200 μm, (**b**) 100 μm

White matter injury was studied by assessing myelin basic protein (MBP) immunoreactivity in the SCWM. Global HI significantly (sham-SAL vs. HI-SAL; *P* < 0.001) reduced MBP immunoreactivity in the SCWM, indicating HI-induced hypomyelination in this region (Fig. 4a–c). MAPC significantly increased MBP immunoreactivity (HI-SAL vs. HI-MAPC; *P* = 0.016). No differences in MBP immunoreactivity in the SCWM were observed between sham animals treated with saline or MAPC (Fig. 4a–c). We determined brain weight as a surrogate for neuronal injury (Table 1). Brain weight was significantly reduced after global HI (sham-SAL vs. HI-SAL; *P* = 0.046). There was no significant difference between saline and MAPC treatment in effect on brain weight (HI-SAL vs. HI-MAPC; *P* = 0.429).

Apoptotic cell death was evaluated in the SCWM by assessment of the number of cleaved Caspase-3-positive cells (Fig. 4d). No differences in numbers of Caspase-3-positive cells were found 7 days after global HI (sham-SAL vs. HI-SAL; *P* = 0.135). The density of apoptotic cells remained unaffected by systemic administration of MAPC (HI-SAL vs. HI-MAPC; *P* = 0.881).

We did not detect MAPC in brain, spleen, or lung tissue 7 days after global HI (i.e., 3 days after the second dose of MAPC) using PCR for human-specific β-2 microglobulin (data not shown).

### MAPC prevented splenic involution and modulated the peripheral inflammatory response

**Fig. 5:**
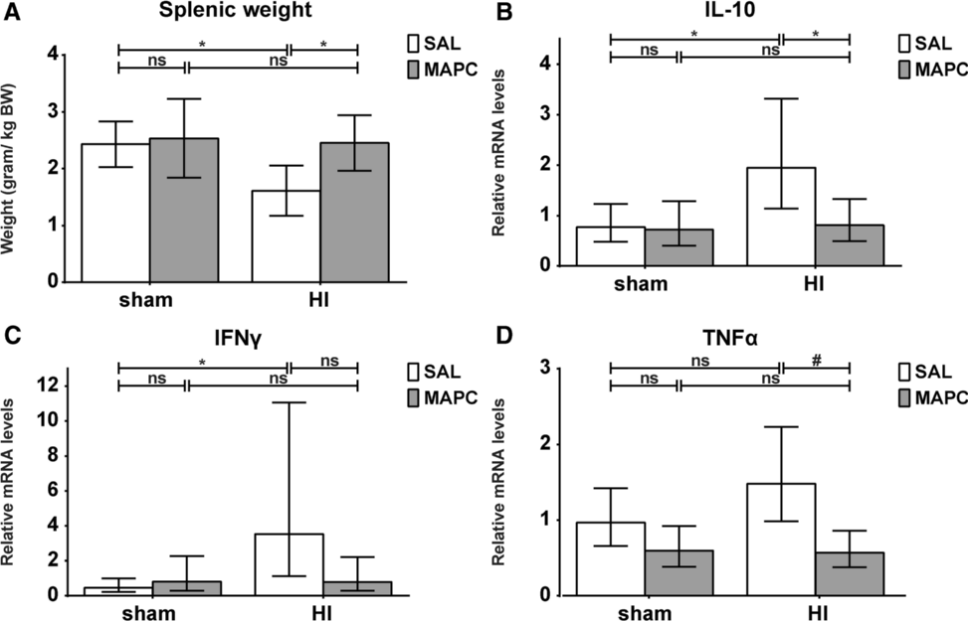
MAPC prevented splenic involution and modulated the peripheral inflammatory response. **a** Splenic weight corrected for body weight (BW). Global HI induced significant splenic involution, which was significantly prevented by MAPC; means ± 95 % CI and levels of significance are depicted, which were calculated with ANOVA (sham-SAL *n* = 8, sham-MAPC *n* = 8, HI-SAL *n* = 8, HI-MAPC *n* = 8). **b** IL-10 mRNA levels were significantly increased in the spleen 7 days after global HI. MAPC significantly attenuated the HI-induced IL-10 response in the spleen. **c** Splenic IFNγ mRNA levels were significantly increased 7 days following global HI. MAPC treatment tended to reduce IFNγ mRNA levels. However, statistical significance was not reached. **d** TNFα mRNA levels showed a trend to increase following global HI, which was significantly prevented by MAPC; geometric means ± 95 % CI and levels of significance are depicted, which were calculated with ANOVA (sham-SAL *n* = 8, sham-MAPC *n* = 6, HI-SAL *n* = 8, HI-MAPC *n* = 8). *P ≤ 0.05, §*P* ≤ 0.01, #*P* ≤ 0.001. *HI* hypoxia-ischemia, *SAL* saline, *MAPC* multipotent adult progenitor cells, *IFNγ* interferon gamma, *TNFα* tumor necrosis factor alpha

Splenic weight was assessed, since activation of the splenic inflammatory response was previously shown to be characterized by mobilization of effector immune cells affecting volume and weight of the spleen [14]. Global HI induced splenic involution in saline-treated animals (sham-SAL vs. HI-SAL; *P* = 0.022) (Fig. 5a). MAPC significantly prevented splenic involution after global HI (HI-SAL vs. HI-MAPC; *P* = 0.032). The splenic cytokine profile was studied since previous findings indicated that MAPC established neuroprotection by modulating the splenic inflammatory response (Fig. 5b–d). Splenic IL-10 mRNA levels were significantly increased after global HI (sham-SAL vs. HI-SAL; *P* = 0.031). MAPC significantly reduced IL-10 mRNA levels in the spleen after global HI (HI-SAL vs. HI-MAPC; *P* = 0.042) (Fig. 5b). IFNγ mRNA levels were significantly increased following global HI (sham-SAL vs. HI-SAL; *P* = 0.022). Systemic administration of MAPC tended to reduce IFNγ mRNA levels, but statistical significance was not reached (HI-SAL vs. HI-MAPC; *P* = 0.078) (Fig. 5c). Global HI tended to increase splenic TNFα mRNA levels (sham-SAL vs. HI-SAL; *P* = 0.078). However, MAPC treatment significantly reduced TNFα mRNA levels (HI-SAL vs. HI-MAPC; *P* < 0.001) (Fig. 5d).

### MAPC reduced seizure burden after global HI

**Fig. 6:**
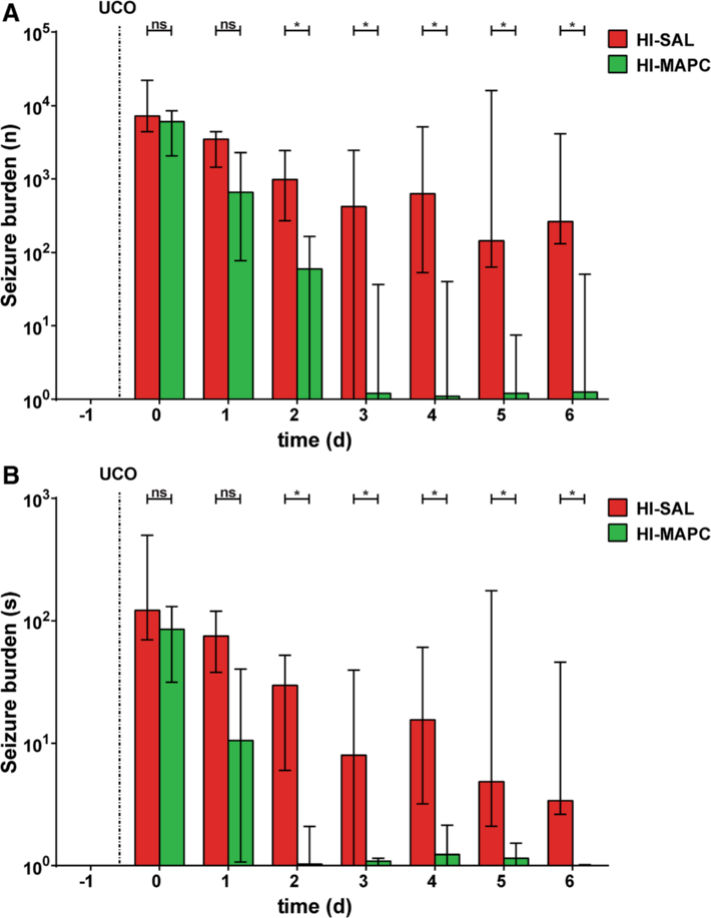
MAPC induced functional neuroprotection after global HI. Global HI caused a significant seizure burden indicated by an increased total number (**a**) and duration of seizures (**b**) compared to controls. Administration of MAPC significantly reduced electrographic seizure number and duration. Medians ± interquartile ranges (IQR) and levels of significance of the treatment effect (HI-SAL vs. HI-MAPC) are depicted, which were calculated by Mann-Whitney test (HI-SAL *n* = 8, HI-MAPC *n* = 8). No electrographic seizure activity was detected under sham conditions. For clarity purposes these sham groups (sham-SAL *n* = 8 and sham-MAPC *n* = 6) are not shown. *P ≤ 0.05, §*P* ≤ 0.01, *#*P ≤ 0.001. *HI* hypoxia-ischemia, *SAL* saline, *MAPC* multipotent adult progenitor cells

Electrographic seizure activity was assessed to determine the effect of MAPC on cortical function after global HI (Fig. 6a–b). Global HI induced a significant increase in total number (Fig. 6a) and duration (Fig. 6b) of seizures after global HI (sham-SAL vs. HI-SAL, significant in all time-points). Seizure burden peaked within the first 48 h following global HI with a second small rise in activity around experimental days 4 and 5 (Fig. 6a–b). MAPC reduced seizure burden throughout the complete reperfusion period (HI-SAL vs. HI-MAPC, significant from experimental day 2 onwards). Cumulative number of seizures in the complete reperfusion study was significantly increased after global HI (sham-SAL vs. HI-SAL; *P* = 0.016) and significantly reduced by MAPC (HI-SAL vs. HI-MAPC; *P* = 0.022) (Fig. 6a). Similarly, cumulative duration (s) of seizures was significantly increased after global HI (sham-SAL v. HI-SAL; *P* = 0.015) and significantly reduced by MAPC (HI-SAL vs. HI-MAPC; *P* = 0.043) (Fig. 6b). No electrographic seizure activity was detected under sham conditions.

### MAPC prevented loss of baroreflex sensitivity after global HI

**Fig. 7:**
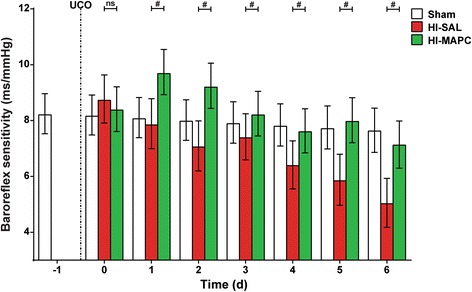
MAPC prevented loss of baroreflex sensitivity. Global HI caused a significant gradual decline of baroreflex sensitivity over time, which was prevented by MAPC treatment; means ± 95 % CI and levels of significance of the treatment effect (HI-SAL vs. HI-MAPC) are depicted, which were calculated by the Bayesian multi-level model. There were no differences in baroreflex sensitivity between the sham-SAL and sham-MAPC groups. For clarity purposes, all sham-treated animals (sham-SAL *n* = 8 and sham-MAPC *n* = 6) were grouped and depicted as one sham group. HI-SAL *n* = 8, HI-MAPC *n* = 8. *P ≤ 0.05, §*P* ≤ 0.01, #*P* ≤ 0.001. *HI* hypoxia-ischemia, *SAL* saline, *MAPC* multipotent adult progenitor cells

Baroreceptor reflex sensitivity was analyzed to study the effect of MAPC treatment on deeper brain functions situated in the brain stem (Fig. 7). Global HI increasingly compromised baroreceptor reflex sensitivity over time (sham-SAL vs. HI-SAL, significant for all time-points with the exception of experimental day 1). MAPC significantly prevented the loss of baroreflex sensitivity (HI-SAL vs. HI-MAPC, significant on all time-points).

## Discussion

Hypoxic-ischemic brain injury is common in preterm infants. To date, no treatment options are available to reduce mortality and morbidity associated with brain injury in preterm infants. Our group has previously shown in a well-established pre-clinical model of global hypoxic-ischemia (HI) that mesenchymal stem cells (MSC) are a promising candidate for the treatment of hypoxic-ischemic brain injury [2]. In the current study, we investigated the neuroprotective capacity of multipotent adult progenitor cells (MAPC), which are adherent bone marrow-derived cells of an earlier developmental stage than MSC and exhibiting more potent anti-inflammatory and regenerative properties [3, 4, 6–10]. We showed that intravenous administration of MAPC prevented functional and structural injury in the preterm brain and modulated the cerebral and peripheral inflammatory response after global HI.

We administered MAPC cells 1 h and 4 days after global HI. With the first dose, we aimed to dampen the acute cerebral and peripheral inflammatory response, which has been shown to evolve early after global hypoxia-ischemia [2]. A pilot study in this model showed a secondary peak in seizure burden in untreated animals 4–5 days after global HI (unpublished data). Therefore, the second dose of MAPC cells was administered 4 days after global HI aimed at preventing secondary functional injury.

Our findings showed that MAPC cells 1 h and 4 days after global HI reduced seizure burden after global HI throughout the study period of 7 days. This finding is clinically highly relevant, since several studies have shown that seizures in term and preterm neonatal HIE are associated with adverse neurodevelopmental outcome [26–28]. In our model, we detected seizure activity with amplitude-integrated EEG (aEEG), which is clinically used to monitor neonatal brain function and has a high sensitivity and specificity in predicting neurodevelopmental outcomes in neonates with HI brain injury [29]. Recorded seizure activity in our study was most likely located in the cortical layers. However, since the electrodes were placed directly on the dura, we may have recorded seizures or electrophysiological responses originating from deeper regions underlying the cortex (i.e., thalamus and hippocampus). In addition to cortical grey matter protection, the reduced seizure activity may therefore also represent protection of deeper brain structures. We therefore assessed the baroreceptor reflex as an indicator for function of the brain stem. This is clinically of importance since the baroreflex is a vital part of the cardiovascular auto regulatory system in preterm infants, which secures adequate perfusion of the preterm brain [22]. The cerebrovascular system is still immature in prematurity, making the preterm brain extremely vulnerable to fluctuations in blood pressure caused by HI (or other adverse events such as infection) [30]. Previously, we demonstrated that global HI disturbed normal development of the baroreflex, which resulted in impairment of heart rate-mediated blood pressure control [22]. The fact that MAPC treatment at 1 h and 4 days improved baroreflex sensitivity indicated that the therapeutic effects reach as far as the highly conserved central brain functions in the brainstem and supports the concept of MAPC-induced protection against grey matter injury in the hypoxic-ischemic preterm brain. The protection of grey matter by MAPC after global HI may be explained by findings of previous reports demonstrating that MAPC improved neurogenesis and stimulated neurite outgrowth and synaptogenesis [7, 31, 32].

Density of apoptotic cells in the subcortical white matter was neither affected by global HI nor by MAPC treatment in this study. The fact that we did not detect significant changes in cell death is most likely explained by timing of assessment at 7 days after global HI, while apoptotic cell death peaks earlier after cerebral hypoxia-ischemia. Further studies on the dynamics of cell death in after global HI are needed to determine the therapeutic effect of MAPC on cellular loss in the preterm brain.

MAPC have been previously shown to have potent anti-inflammatory properties in pre-clinical studies of traumatic and ischemic injury of the central nervous system (CNS) [6–11]. Our study confirmed and extended these findings in a translational model of hypoxic-ischemic injury in the preterm brain by showing that MAPC reduced the neuroinflammatory response in the subcortical white matter (SCWM), as evidenced by reduced microglial proliferation. We and others have shown that the microglial response is a reliable marker for the cerebral inflammatory response that plays a pivotal role in the etiology of hypoxic-ischemic encephalopathy in the preterm brain [14, 33]. Although the size of the treatment effect was modest, reduction of IBA-1 immunoreactivity was associated with functional and structural improvement, indicating that the modulation of the microglial response was biologically relevant.

We postulate that the observed MAPC-induced reduction of cerebral inflammation after global HI may be explained by two mechanisms; firstly, MAPC have been shown to induce a microglial-type switch from a pro-inflammatory M1 phenotype to an anti-inflammatory M2 phenotype, which is associated with reduced inflammation in the CNS [8]. Secondly, MAPC may have reduced glutamate excitotoxicity in a similar fashion as MSC, which were shown previously to inhibit glutamate receptor expression and function, thereby inhibiting microglial activation [34]. However, MAPC did not attenuate the inflammatory response in the hippocampus. These spatial differences in anti-inflammatory effects are in line with previous data showing that the hippocampus is more severely affected by hypoxia-ischemia, and therefore, injury may be irreversible [2, 14, 15].

White matter injury is the clinical hallmark of HI brain injury in the preterm brain [35]. Consistent with earlier studies [2, 14, 15], we demonstrated that global hypoxia-ischemia induced marked hypomyelination of the preterm brain, which was prevented by intravenous administration of MAPC. Microglial-type switch and subsequent reduction of cerebral inflammation has been previously associated with remyelination [36, 37] and may therefore explain the observed reduction of white matter injury.

Besides attenuation of the cerebral inflammatory response, our findings indicated that MAPC modulated the peripheral inflammatory response, which has been implicated in the etiology of hypoxic-ischemic injury in the preterm brain [14, 38–40]. Our results showed that MAPC prevented splenic involution. We have previously shown in the current model that splenic involution after global HI was caused by efflux of immune effector cells [14]. Furthermore, our findings demonstrated that intravenous administration of MSC induced splenic T cell tolerance [2]. The observed unresponsiveness of splenic T cells was associated with reduced systemic mobilization of immune effector cells and concomitant reduction of brain-infiltrating immune cells resulting in less severe inflammation in the preterm brain after global HI [2]. Based on these findings, we postulate that MAPC therapy reduced the mobilization of immune effector cells from the spleen resulting in less extensive invasion of these injurious cells into the brain. This concept is supported by previous findings showing a spleen-mediated neuroprotective effect of MAPC in traumatic CNS injury [9, 11]. Recently, it was demonstrated that splenic IFNγ plays a key role in injurious signaling to the ischemic brain [41]. Functional elimination by splenectomy or administration of IFNγ neutralizing antibodies reduced brain injury after stroke [42]. In line with these findings, reduction of cerebral inflammation (i.e., microglial proliferation) by MAPC treatment was associated with decreased splenic IFNγ levels. To determine whether IFNγ from spleen cells is involved in preterm brain injury after global HI, temporal dynamics of splenic IFNγ release needs to be elucidated and its functional role to be determined.

In our study, splenic IL-10 mRNA levels were not altered by MAPC in sham conditions. Instead, we found a marked increase of IL-10 mRNA under hypoxic-ischemic conditions, which was attenuated by administration of MAPC. We postulate that the observed increase of splenic IL-10 mRNA after global HI is part of an endogenous regenerative response after injury. MAPC may have reduced the injurious load eliciting such a response resulting in reduced IL-10 levels at the time of assessment (7 days after global HI). This hypothesis is supported by previous reports showing activation of endogenous repair processes involving IL-10-mediated T-reg mobilization after cerebral ischemia [39, 40, 43]. Furthermore, early administration of MSC was shown to prevent progression of hypoxic-ischemic injury [2]. These findings suggest that early administration of MAPC prevent injury before endogenous repair mechanisms, such as IL-10-mediated T-reg mobilization, are activated.

In line with previous studies that report the clearance of MAPC within 48–72 h [10, 31], we did not detect MAPC in brain, spleen, or lung tissue 7 days after global HI (i.e., 3 days after the second dose of MAPC). The fact that we found MAPC-mediated therapeutic effects without detectable presence of MAPC in the brain after 7 days of onset of hypoxia-ischemia, suggests that the neuroprotective effects of MAPC last longer than their presence. We postulate that this phenomenon can be explained by MAPC dampening the detrimental, peripheral, and cerebral inflammatory responses at an early stage, before these responses can aggravate hypoxic-ischemic injury in the preterm brain.

Our study has several limitations. We did not perform a time series of histologic analysis to assess temporal dynamics of global HI-induced injury, the cerebral and peripheral inflammatory response, and therapeutic effects of intravenous MAPC administration. Moreover, we did not discriminate between the therapeutic effect between the first and second dose of MAPC cells. These limitations are inherent to limited experimental groups to make large animal experiments feasible. Unfortunately, no ovine-specific antibodies are currently available to detect M1 and M2 microglial phenotypes in our studies.

Our findings are clinically highly relevant for several reasons. First, we tested our hypothesis in a well-established pre-clinical animal in which global HI was induced by transient umbilical cord occlusion accurately mimicking the common etiology of hypoxic-ischemic brain injury in preterm infants [44, 45]. Second, this pre-clinical animal model allows for continuous in utero registration of relevant parameters (i.e., vital parameters and aEEG) with strong clinical relevance and predictive value, which gives more insight in HI-induced pathophysiology and efficacy of therapeutic options in a pre-clinical setting. Third, pre-clinical studies have confirmed safety and genomic stability of MAPC [5, 23], and MAPC therapy is currently being tested in phase I/II clinical trials for the treatment of ischemic stroke, graft-versus-host disease (GVHD), acute myocardial infarction, and inflammatory bowel disease [4], emphasizing clinical applicability of these cells.

## Conclusions

In conclusion, we have shown in a pre-clinical animal model that MAPC improve the functional and structural outcome of the preterm brain after global HI. Future studies should further establish the mechanism and long-term therapeutic effects of MAPC-induced neuroprotection in the developing preterm brain exposed to hypoxia-ischemia. Our study may form the basis for future clinical trials, which will evaluate whether MAPC therapy is capable of reducing neurological sequelae in preterm infants with hypoxic-ischemic brain injury.

## Abbreviations

aEEG: amplitude-integrated electroencephalogram
ANOVA: analysis of variance
BW: body weight
CD: cluster of differentiation
CI: confidence interval
CNS: central nervous system
ECG: electrocardiogram
EEG: electroencephalogram
FDR: false discovery rate
FHR: fetal heart rate
GA: gestational age
GVHD: graft-versus-host disease
H_2_O_2_: hydrogen peroxide
HI: hypoxia-ischemia
HIE: hypoxic-ischemic encephalopathy
IBA-1: ionized calcium-binding adaptor molecule 1
IL: interleukin
IFNγ: interferon gamma
IQR: interquartile range
IR: immunoreactivity
MABP: mean arterial blood pressure
MAPC: multipotent adult progenitor cells
MBP: myelin basic protein
MHC: major histocompatibility complex
MPAQ: Maastricht-Programmable AcQuisition system
MSC: mesenchymal stem cells
ovRPS15: ovine 40S ribosomal protein S15
RT-qPCR: real-time quantitative polymerase chain reaction
SAL: saline
SBP: systolic blood pressure
SD: standard deviation
SCWM: subcortical white matter
SD-RR: standard deviation of the mean R-R interval
SD-SBP: standard deviation of the mean systolic blood pressure
TBS: tris-buffered saline
TNFα: tumor necrosis factor alpha
UCO: umbilical cord occlusion
